# Impact of HIV and hospitalization on the incidence of subsequent rifampicin-resistant tuberculosis after initiation of first-line tuberculosis treatment: a retrospective cohort study in South Africa

**DOI:** 10.1016/j.eclinm.2025.103603

**Published:** 2025-10-30

**Authors:** N. Zinyakatira, M. Smith, A. Boulle, N. Tiffin, L.F. Johnson, N. Ford, H. Cox

**Affiliations:** a Centre for Integrated Data and Epidemiological Research, School of Public Health, University of Cape Town, South Africa; b Division of Medical Microbiology, Faculty of Health Sciences, University of Cape Town, South Africa; c Division of Public Health Medicine, School of Public Health, University of Cape Town, South Africa; d Department of Health, Health Intelligence, Western Cape Government, South Africa; e South African National Bioinformatics Institute, University of the Western Cape, South Africa; f Burnet Institute, Melbourne, Australia

**Keywords:** Human immunodeficiency virus, Drug resistant tuberculosis, Rifampicin, Tuberculosis treatment, Resistance-acquisition, Nosocomial transmission

## Abstract

**Background:**

People living with human immunodeficiency virus (PLHIV) may have a higher risk of acquired rifampicin-resistance during first-line tuberculosis (TB) treatment, potentially driving the multi-drug or rifampicin-resistant tuberculosis (MDR/RR-TB) epidemic. Nosocomial transmission may further elevate MDR/RR-TB risk. We assessed the impact of HIV and hospitalization on subsequent MDR/RR-TB diagnosis among individuals starting first-line TB treatment.

**Methods:**

The retrospective cohort included individuals with laboratory-confirmed rifampicin-susceptible TB (RS-TB), who started TB treatment (2013–2021). Subsequent TB diagnoses (MDR/RR-TB and RS-TB) over 2 years’ follow-up from TB treatment initiation were assessed. Routine health service data utilized.

**Findings:**

A total of 190,945 individuals were included; median age 34.0 (interquartile range (IQR), 25.5–44.9); 79,160 (42%) female and 69,636 (37%) PLHIV. Overall, 6870 (9.9%) PLHIV and 9342 (7.7%) HIV-negative individuals were diagnosed with recurrent TB within 24 months. Rifampicin drug susceptibility testing was available for 5354 (77.9%) and 8154 (87.3%) PLHIV and HIV-negative individuals, respectively. PLHIV with advanced HIV (cluster of differentiation 4 (CD4) <200 cells/μl) (adjusted-hazard ratio (HR) 2.86, 95% confidence interval (CI), 2.60–3.15) and individuals hospitalized (adjusted-HR 2.76, 95% CI, 2.50–3.05) for ≥1 week had significantly increased MDR/RR-TB risk compared to HIV-negative and non-hospitalized individuals, respectively. PLHIV had a higher risk of MDR/RR-TB relative to all other recurrent TB, regardless of CD4.

**Interpretation:**

This study suggests that PLHIV may have an increased risk of both acquiring rifampicin-resistance during TB treatment and re- or super-infection with already resistant *Mycobacterium tuberculosis* strain during hospitalization. While not causal, these data suggest the need for improved TB treatment for PLHIV including tailored drug regimens, potentially with increased rifampicin dosages, and emphasize the importance of TB infection control in healthcare settings.

**Funding:**

The study received no funding.

**Research in context**

**Evidence before this study**

Modelling studies suggest that while direct transmission of already resistant *Mycobacterium tuberculosis* strains is a key driver of the multi-drug or rifampicin-resistant tuberculosis (MDR/RR-TB) epidemic, resistance that emerges during tuberculosis (TB) treatment (acquired resistance) will remain a significant contributor. However, the extent to which acquired resistance is driving the MDR/RR-TB epidemic is unknown, especially in high human immunodeficiency virus (HIV) prevalence settings. Additionally, the contribution of nosocomial transmission to the MDR/RR-TB epidemic may be significant, particularly for people living with HIV (PLHIV), given that both people with infectious MDR/RR-TB and immunocompromised individuals use the same healthcare facilities. We searched PubMed, EBSCOhost, Scopus, and Web of Science for English-language studies published up to the 31st of May 2025. We reviewed studies that investigated the association between MDR/RR-TB, HIV, hospitalization, resistance-acquisition and transmission. There is evidence that TB drug resistance continues to be diagnosed during TB treatment, with an increased risk among PLHIV, particularly those with advanced HIV, compared to HIV-negative individuals. This increased risk could be due to a higher risk of resistance-acquisition during TB treatment or an increased risk of transmission (potentially nosocomial) of MDR/RR-TB strains, or both. However, no large-scale studies have quantified the effects of HIV (and related immunosuppression) and hospitalization on the risk of MDR/RR-TB diagnosis during and after TB treatment in high TB and HIV burden settings.

**Added value of this study**

This is the first study of a large cohort in a high TB and HIV burden setting quantifying the association of HIV and hospitalization with subsequent MDR/RR-TB diagnosis in individuals starting first-line TB treatment. This cohort represents about 95% of individuals with HIV treated for TB disease in the Western Cape Province, South Africa, making findings generalizable to similar populations. We have shown that PLHIV who initiate treatment for drug-susceptible TB have a significantly higher risk of subsequent diagnosis with MDR/RR-TB compared to HIV negative individuals. This increased risk is particularly evident among those with advanced HIV disease. Additionally, individuals hospitalized for more than a week had a substantially increased risk of subsequent MDR/RR-TB diagnosis compared to non-hospitalized individuals.

**Implications of all the available evidence**

These data suggest that PLHIV may require differentiated TB treatment involving tailored drug regimens with increased rifampicin doses and intensified monitoring for treatment response to minimize the risk of TB drug resistance-acquisition during treatment. Individualized TB treatment for PLHIV that considers the degree of immunocompromise, TB disease severity, other comorbidities and body weight may be required. This is particularly relevant given recent and current TB treatment trials testing shorter TB treatment durations, which may further increase the risk of resistance-acquisition. While such trials are unlikely to be powered to detect resistance emergence as an outcome, they should include embedded pharmacokinetic studies with sufficient numbers of PLHIV. South Africa's generalized HIV and TB epidemics characterized by widespread transmission in the general population, together with socio-economic factors and other unmeasured contributors, may also increase MDR/RR-TB risk, highlighting the need to include these factors in future research to assess their impact on MDR/RR-TB diagnosis among PLHIV. Finally, while the risk of TB transmission in healthcare settings is well known, these data again emphasize the need for effective TB infection control measures.

## Introduction

Globally, the number of individuals estimated to develop multi-drug or rifampicin-resistant tuberculosis (MDR/RR-TB) has remained at approximately half a million annually over the past decade.^1^ While modelling studies suggest that direct transmission is the predominant epidemic driver in high burden countries, likely due to substantial gaps in the MDR/RR-TB care continuum,^2^ there is also evidence suggesting that tuberculosis (TB) drug resistance continues to emerge *de novo* during treatment.^3^ MDR/RR-TB is strongly associated with previous first-line treatment, reflecting acquired resistance under treatment pressure.^4^ While this has often been attributed to poor treatment adherence, empirical evidence suggests that other factors such as individual-level pharmacokinetics may be more important.^5^ Reducing both transmission of already resistant TB strains and acquired resistance during treatment is key to addressing the MDR/RR-TB epidemic.

Systematic reviews suggest that human immunodeficiency virus (HIV) is associated with MDR/RR-TB.^3^^,^^6^^,^^7^ This association could be driven through either increased acquired resistance during treatment or higher MDR/RR-TB infection risk relative to drug-susceptible TB (DS-TB). Increased MDR/RR-TB infection among people living with HIV (PLHIV) could result from a disproportional influence of nosocomial transmission, particularly in high HIV prevalence settings where exposure to MDR/RR-TB and increased vulnerabilities (including HIV immunosuppression and other comorbidities) co-exist in healthcare facilities.^8^^,^^9^ HIV has also been specifically associated with acquired MDR/RR-TB during treatment,^3^ an association that could be due to HIV-related pharmacokinetic effects, potentially through malabsorption of TB drugs,^10^ an effect more likely for rifampicin.^9^^,^^11^^,^^12^

South Africa is ranked among the 30 highest TB-burden countries globally, with an estimated TB incidence of 468 per 100,000 annually and HIV co-infection of 54%.^1^ Among the 280,000 individuals who develop TB annually, 11,000 have MDR/RR-TB. While the extent to which acquired MDR/RR-TB during treatment is driving the MDR/RR-TB epidemic in South Africa is unknown, modelling suggests that acquired MDR/RR-TB will remain a significant contributor.^13^ To date this modelling has not considered increased acquired MDR/RR-TB risk among PLHIV.

We have previously shown that individuals diagnosed with MDR/RR-TB, who were known to be HIV-positive during prior TB treatment, were less likely to be infected with MDR/RR-TB strains in genomic clusters compared to HIV-negative individuals with prior TB treatment.^14^ These previous study results suggest that MDR/RR-TB among PLHIV with prior TB treatment may be more likely to be due to resistance-acquisition during that prior treatment and not directly transmitted MDR/RR-TB. We therefore hypothesized that MDR/RR-TB resistance-acquisition during treatment is more likely among PLHIV compared to HIV-negative individuals. To test this hypothesis, using large scale-routine data, we assessed the impact of HIV and hospitalization on the risk of subsequent RR-TB diagnosis among individuals who were treated for rifampicin-susceptible TB (RS-TB) disease in the Western Cape Province (WCP), South Africa.

## Methods

### Study design

This was a retrospective cohort study utilizing anonymized routine health data from public health facilities in WCP, South Africa. The primary outcome was the risk of incident MDR/RR-TB over a 2-year period following first-line TB treatment initiation among PLHIV and HIV-negative individuals diagnosed with RS-TB.

### Study setting and data sources

The WCP has an estimated population of 7.4 million. While approximately 75% of the population utilize public healthcare services overall, over 95% utilize the public sector for all or most of their HIV or TB care.^15^

South African TB guidelines include routine drug susceptibility testing (DST) to rifampicin using the WHO recommended rapid molecular diagnostic tests (Xpert *M. tuberculosis*/rifampicin (MTB/RIF)) for all individuals investigated for TB. The Provincial Health Data Centre (PHDC), within the WCP Department of Health, integrates routine patient-level data from multiple health information systems including disease management, pharmacy, hospital, clinic, and laboratory systems using a unique patient identifier.^15^ A de-identified dataset was extracted from the PHDC for this study on March 7, 2024.

### Cohort inclusion criteria

The cohort included all individuals (regardless of age) who commenced first-line TB treatment between January 1, 2013, and December 31, 2021, with a laboratory-confirmed first RS-TB diagnosis. The RS-TB diagnosis was defined by a RS-TB result falling within 6 months prior, or up to 2 weeks after, treatment initiation. Participants were followed up through the PHDC to determine subsequent TB diagnoses (incident TB) within 2 years of treatment initiation. Any recurrent TB diagnosis was based on evidence that included: laboratory results, provision of TB treatment, TB international classification of diseases, 10th edition (ICD-10) code, TB hospital admission and registration in TB registers. However, subsequent MDR/RR-TB relied solely on laboratory DST data.

PLHIV were defined as individuals who were known to be HIV-positive either before or within 3 months of starting TB treatment. Given that HIV-negative status is not directly recorded, HIV-negative individuals were defined as those without evidence of HIV from laboratory testing (cluster of differentiation 4 (CD4) cell count, viral load, positive HIV test), or evidence of antiretroviral treatment (ART), or HIV ICD-10 code (for any healthcare interaction) or registration in the HIV register. For PLHIV, CD4 cell counts closest to TB treatment initiation were included, within a 12-month period from 6 months before to 6 months after TB treatment initiation. Although routine CD4 count monitoring is no longer standard-of-care for virologically suppressed patients on ART, CD4 counts are usually available in PLHIV who are acutely unwell or newly diagnosed with TB. According to South African treatment guidelines, all PLHIV diagnosed with TB and in care are expected to be on ART, with immediate initiation if not already on it. CD4 cell count was therefore included in the analyses as a general marker of HIV disease status, reflecting both any effects of duration on, and interruptions to, ART.

We calculated cumulative duration of hospital admission as a time-updated covariate across both a 6-month period after treatment initiation and between the TB treatment outcome date and either subsequent MDR/RR-TB diagnosis, censoring, or within an 18-month period from the outcome date for individuals with no subsequent TB diagnosis. The successful treatment outcomes were defined as either “completed” or “cured” as recorded in the TB register, according to World Health Organization (WHO) definitions.

### Statistical analysis

Parametric survivor (time-to-event) models, assuming a Gompertz distribution for the survivor probabilities were used to assess the impact of HIV (and HIV severity, assessed by CD4 cell count) on the incidence of subsequent MDR/RR-TB (DST) from the timepoint of first-line TB treatment initiation. The Gompertz distribution was identified as the best fit based on the Akaike Information Criterion (AIC) (see Supplementary Information). Other risk factors included in the models, defined *a priori* were sex, age and cumulative hospital admission (defined above as a time-updated covariate). A hypothetical visual pathway has been included in supplementary (Supplementary Figure S1). To further assess the potential impact of nosocomial transmission and other comorbidities not able to be measured, and to negate the effect of hospital admission due to poor outcomes (TB treatment failure, poor adherence and loss-to-follow-up), we conducted separate analyses by HIV-status among individuals who were recorded as successfully treated. Kaplan-Meier curves were plotted to compare survival times for categories of CD4 cell count. We assessed the goodness of fit of the selected Gompertz model using the Cox-Snell residuals (Supplementary Tables S3, S4, Supplementary Figure S3). In addition, we used the variance inflation factor (VIF) to detect multicollinearity among the risk factors (Supplementary Table S5).

The data were censored for death and loss-to-follow-up based on the last health care system activity date. Given that MDR/RR-TB observed more than 2 years after treatment is less likely to represent resistance-acquisition, a 2-year follow-up period was used. To assess the impact of hospitalization among individuals with treatment success, an 18-month follow-up from treatment outcome was used.

Additionally, we assessed whether HIV (and CD4 cell count) was differentially associated with the incidence of MDR/RR-TB compared to all other diagnosed TB (including empiric TB diagnosis), by conducting separate analyses with the incidence of MDR/RR-TB and all other TB (non-MDR-/RR-TB) as the outcomes. This analysis was restricted to successfully treated individuals with follow-up of 18 months from TB treatment outcome date. Statistical analyses were performed using Stata, version 15.1.

### Ethics

This study was approved by the University of Cape Town, Human Ethics Research Committee (HREC) (HREC reference number 222/2022), and the WCP Health Research Committee (WCPHRC reference number WC_202204_035). A waiver of informed consent was granted by the HREC, as researchers only received de-identified data.

### Role of the funding source

The study received no funding.

## Results

### Cohort description

**Fig. 1:**
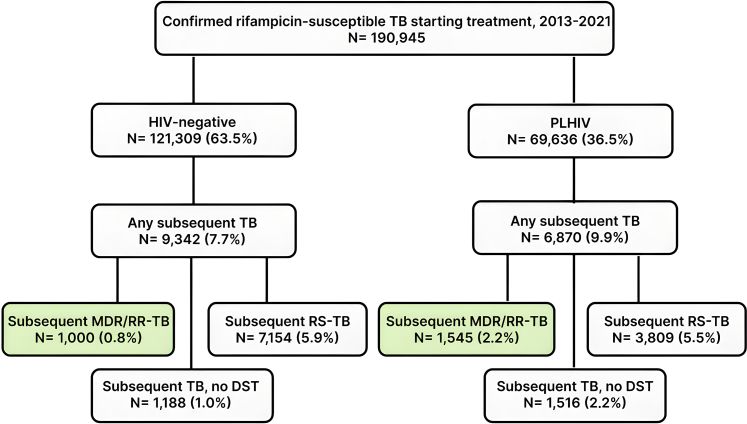
**Flowchart of study cohort and subsequent tuberculosis (TB) episodes within 24 months of treatment start.** Abbreviations: Human immunodeficiency virus (HIV); people living with HIV (PLHIV); multi-drug or rifampicin-resistant tuberculosis (MDR/RR-TB); rifampicin-susceptible TB (RS-TB); drug susceptibility testing (DST).

Overall, 248,514 individuals were diagnosed with microbiologically confirmed RS-TB between 2013 and 2021; of these, 57,569 (23.2%) were excluded as treatment initiation could not be confirmed. Therefore, 190,945 individuals were included in the cohort; 69,636 (37%) PLHIV, the median age was 34 years, and 42% were female (Fig. 1, Table 1). PLHIV were more likely to be older, males, have received TB treatment in the Cape Town metro versus other districts and been admitted to hospital within 6 months of treatment initiation (Table 1, Supplementary Table S1). Additionally, 91% of PLHIV had evidence of starting ART, with 80% having evidence of being on ART at TB treatment initiation. The median CD4 cell count for PLHIV was 133 (interquartile range (IQR), 56–284) cells/μl, and 13% did not have a CD4 cell count available. Notably, 55% of PLHIV had advanced HIV disease (CD4 cell count below 200 cells/μl).

**Table 1:** Baseline description of the cohort (N = 190,945) by HIV status at first-line TB treatment initiation.

	HIV-negative (%)	PLHIV (%)	Total (%)
Total	121,309	69,636	190,945
Female	45,067 (37.2)	34,093 (49.0)	79,160 (41.5)
Median age, IQR	32.6 (22.9–47.3)	35.2 (29.3–42.2)	34.0 (25.5–44.9)
Age group			
0–14	8048 (6.6)	846 (1.2)	8894 (4.7)
15–24	29,788 (24.6)	6709 (9.6)	36,497 (19.1)
25–34	28,736 (23.7)	26,765 (38.4)	55,501 (29.1)
35–44	19,906 (16.4)	22,722 (32.6)	42,628 (22.3)
45–54	18,366 (15.1)	9519 (13.7)	27,885 (14.6)
55+	16,465 (13.6)	3075 (4.4)	19,540 (10.2)
Year started TB treatment			
2013–2015	39,177 (32.3)	24,764 (35.6)	63,941 (33.5)
2016–2018	42,091 (34.7)	24,078 (34.6)	66,169 (34.7)
2019–2021	40,041 (33.0)	20,794 (29.9)	60,835 (31.9)
TB treatment district			
Cape Town metro	69,039 (56.9)	46,214 (66.4)	115,253 (60.4)
Other districts^a^	52,270 (43.1)	23,422 (33.6)	75,692 (39.6)

Abbreviations: Human immunodeficiency virus (HIV); people living with HIV (PLHIV); interquartile range (IQR); tuberculosis (TB); percentage (%).

a Includes Cape Winelands, Central Karoo, Garden Route, Overberg and West Coast districts.

Individuals with advanced HIV were more likely to be male and younger (Supplementary Table S1). PLHIV were more likely to be hospitalized (32%) and for more than a week (47% among those hospitalized) versus HIV-negative individuals (18% and 36% respectively) (p < 0.01) (Table 2). HIV-negative individuals were more likely to have a successful (completed or cured) treatment outcome compared to PLHIV (79% and 69% respectively) (p < 0.01). Among those with successful TB treatment outcomes, 12% of PLHIV and 9% HIV-negative individuals were hospitalized either before subsequent MDR/RR-TB, censoring or within 18 months of TB treatment outcome date. Of those hospitalized 36% of PLHIV and 29% of HIV-negative individuals were hospitalized for more than a week in total.

**Table 2:** Hospital admission and treatment outcomes by HIV status.

	HIV-negative (%)	PLHIV (%)	Total (%)
Hospital admission within 6 months of treatment initiation	21,433 (17.7)	22,395 (32.2)	43,828 (23.0)
Cumulative admission time			
<1 week	13,765 (64.2)	11,843 (52.9)	25,608 (58.4)
≥1 week	7668 (35.8)	10,552 (47.1)	18,220 (41.6)
Treatment outcome			
Successful treatment^a^	95,492 (78.7)	47,953 (68.9)	143,445 (75.1)
Died	5031 (4.1)	6175 (8.9)	11,206 (5.9)
Unsuccessful outcome^b^	20,786 (17.1)	15,508 (22.3)	36,294 (19.0)
Total	121,309	69,636	190,945
Hospital admission post-successful treatment outcome	8957 (9.4)	5655 (11.8)	14,612 (10.2)
Cumulative admission time			
<1 week	6368 (71.1)	3631 (64.2)	9999 (68.4)
≥1 week	2589 (28.9)	2024 (35.8)	4613 (31.6)

Abbreviations: Human immunodeficiency virus (HIV); people living with HIV (PLHIV); percentage (%).

a Includes both completed treatment and cured.

b Includes LTF, transferred out, moved out of province/other country, unsuccessful treatment (including treatment failure) and unknown.

### Subsequent TB diagnosis

Overall, 6870 (9.9%) PLHIV and 9342 (7.7%) HIV-negative individuals had a subsequent TB diagnosis within 2 years of treatment initiation (p < 0.01) (Fig. 1). The median time to subsequent TB diagnosis was similar for both PLHIV and HIV-negative individuals; 12.4 (IQR, 8.6–17.5) and 12.3 (IQR, 8.9–17.3) months respectively. Rifampicin DST was available for 5354 (77.9%) PLHIV and 8154 (87.3%) HIV-negative individuals with subsequent TB diagnoses (p < 0.01).

### Association between HIV and incidence of subsequent MDR/RR-TB

**Fig. 2:**
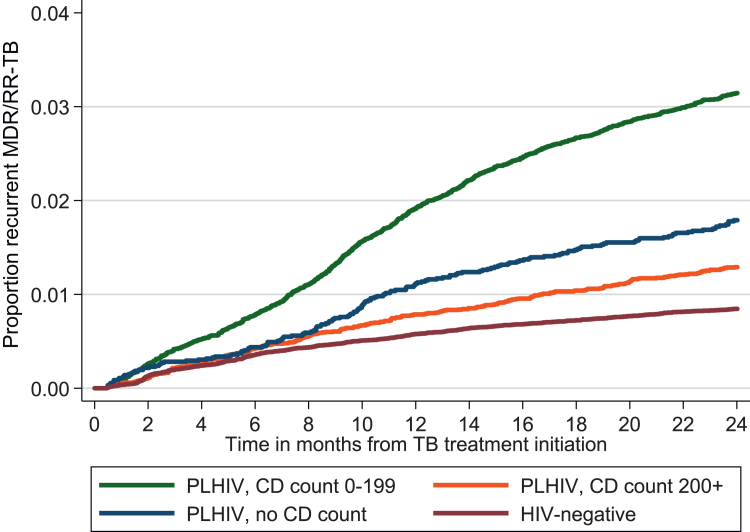
**Kaplan-Meier graphs of subsequent multi-drug or rifampicin-resistant tuberculosis (MDR/RR-TB) by human immunodeficiency virus (HIV) (and CD4 cell count) status within 24 months of treatment start.** Abbreviations: People living with HIV (PLHIV); cluster of differentiation 4 (CD4).

Among PLHIV, 2.2% (1545/69,636) were diagnosed with a subsequent MDR/RR-TB episode within 2 years, compared to 0.8% (1000/121,309) of HIV-negative individuals (Fig. 1), corresponding to a crude odds ratio (OR) of 2.73 (95% confidence interval (CI), 2.52–2.96). In the adjusted Gompertz survivor regression model, PLHIV at all CD4 cell count levels, male individuals, those aged 25–44 years, and those with hospital admissions all had higher risks of MDR/RR-TB over time versus reference categories (Table 3). At 12 and 24 months after treatment initiation, PLHIV with advanced HIV disease had 2.3 and 2.9 times the risk of MDR/RR-TB, respectively, consistently higher than PLHIV with CD4 cell counts ≥200 cells/μl and HIV negative individuals (Fig. 2).

**Table 3:** Univariable and multivariable Gompertz survivor regression models describing the association between HIV (and CD4) with subsequent multi-drug or rifampicin-resistant tuberculosis (MDR/RR-TB) diagnosis.

	Unadjusted HR (95% CI)	Adjusted HR (95% CI)
Exposure of interest		
CD4 cell count ≤±6 months of treatment start		
0–199	3.74 (3.43–4.07)	2.86 (2.60–3.15)
200+	1.53 (1.34–1.74)	1.38 (1.20–1.58)
CD4 missing	2.12 (1.79–2.50)	2.02 (1.71–2.39)
HIV-negative	1 (reference)	1 (reference)
Known potential confounders		
Sex		
Female	1 (reference)	1 (reference)
Male	0.99 (0.92–1.07)	1.10 (1.01–1.19)
Age groups		
0–14	0.44 (0.35–0.57)	0.61 (0.47–0.79)
15–24	0.63 (0.55–0.71)	0.97 (0.85–1.10)
25–34	0.97 (0.88–1.07)	1.04 (0.94–1.15)
35–44	1 (reference)	1 (reference)
45–54	0.71 (0.62–0.81)	0.85 (0.75–0.98)
55+	0.44 (0.37–0.53)	0.63 (0.52–0.76)
Cumulative admission time^a^		
No admission	1 (reference)	1 (reference)
<1 week	1.42 (1.26–1.59)	1.21 (1.07–1.36)
≥1 week	3.61 (3.28–3.96)	2.75 (2.49–3.04)
Constant		0.00050 (0.00044–0.00057)
gamma		−0.0376 (−0.0434 to −0.0319)

Abbreviations: Human immunodeficiency virus (HIV); cluster of differentiation 4 (CD4); hazard ratio (HR); confidence interval (CI).

a Within 6 months of treatment initiation.

### Assessing the impact of hospitalization by HIV-status on subsequent MDR/RR-TB diagnosis

In a subset Gompertz regression analysis among PLHIV individuals with treatment success, those hospitalized for more than a week were 4.2 times more likely to develop MDR/RR-TB compared to those not hospitalized, while those hospitalized for less than a week were 1.8 times more likely (Table 4). Among HIV-negative individuals with treatment success, those hospitalized for more than a week were 2.9 times more likely to be diagnosed with MDR/RR-TB compared to those not hospitalized, with no significant difference observed among those hospitalized for less than a week.

**Table 4:** Multivariable Gompertz survivor regression models describing the association between hospitalization with subsequent multi-drug or rifampicin-resistant tuberculosis (MDR/RR-TB) diagnosis among individuals who successfully completed treatment by HIV status.

	Adjusted HR (95% CI) HIV-negative	Adjusted HR (95% CI) PLHIV	Adjusted HR (95% CI) Overall
Exposure of interest			
Cumulative admission time^a^			
No admission	1 (reference)	1 (reference)	1 (reference)
<1 week	1.42 (0.98–2.06)	1.78 (1.40–2.28)	1.69 (1.38–2.07)
≥1 week	2.92 (1.96–4.33)	4.18 (3.37–5.18)	3.93 (3.25–4.74)
Known potential confounders			
CD4 cell count ≤±6 months of treatment start			
0–199	–	2.61 (2.13–3.20)	4.58 (3.96–5.30)
200+	–	1 (reference)	1.79 (1.44–2.21)
CD4 missing	–	1.55 (1.16–2.08)	2.69 (2.08–3.48)
HIV-negative	–	–	1 (reference)
Sex			
Female	1 (reference)	1 (reference)	1 (reference)
Male	1.17 (0.94–1.46)	1.26 (1.08–1.48)	1.20 (1.06–1.37)
Age groups			
0–14	0.35 (0.17–0.70)	0.68 (0.25–1.83)	0.44 (0.25–0.77)
15–24	0.68 (0.49–0.95)	1.58 (1.22–2.04)	1.03 (0.84–1.26)
25–34	0.93 (0.68–1.26)	1.16 (0.98–1.39)	1.10 (0.94–1.28)
35–44	1 (reference)	1 (reference)	1 (reference)
45–54	1.11 (0.80–1.53)	0.68 (0.52–0.91)	0.88 (0.72–1.08)
55+	0.84 (0.58–1.22)	0.28 (0.13–0.60)	0.68 (0.51–0.92)
Constant	0.0003 (0.0002–0.0004)	0.0005 (0.0004–0.0006)	0.00028 (0.00022–0.00034)
gamma	−0.04 (−0.06 to −0.02)	−0.05 (−0.07 to −0.04)	−0.05 (−0.06 to −0.04)

Abbreviations: Human immunodeficiency virus (HIV); hazard ratio (HR); confidence interval (CI); cluster of differentiation 4 (CD4).

a Within either subsequent MDR/RR-TB diagnosis, censoring or 18 months (if no subsequent TB diagnosis) of TB outcome date.

### Association between HIV and incidence of subsequent MDR/RR-TB compared to RS-TB

To further assess any increased risk of MDR/RR-TB among PLHIV, we compared the cumulative risk of MDR/RR-TB over time with that of all other recurrent TB (Table 5). Among the 6311 PLHIV with recurrent TB, 679 (10.8%) of these were MDR/RR-TB, while among the 9115 HIV-negative individuals with recurrent TB, 369 (4%) of these were MDR/RR-TB. PLHIV had a higher risk of MDR/RR-TB compared to RS-TB for all CD4 cell count categories, with the highest risk observed in the lowest CD4 cell counts. While hospital admission was a significant predictor for both RS-TB and MDR/RR-TB, a higher risk was only observed for MDR/RR-TB for cumulative admission of more than a week (Table 5).

**Table 5:** Multivariable Gompertz survivor regression models describing the incidence of multi-drug or rifampicin-resistant tuberculosis (MDR/RR-TB) compared to all other diagnosed TB (non-MDR/RR-TB) in individuals who successfully completed treatment (18 months’ follow-up from TB outcome date).

	MDR/RR-TB Adjusted HR (95% CI)	All other diagnosed (recurrent) TB Adjusted HR (95% CI)	Differential risk RR-TB/RS-TB
Exposure of interest			
CD4 cell count ≤±6 months of treatment start			
0–199	1.38 (1.32–1.44)	4.58 (3.96–5.30)	3.33
200+	1.10 (1.04–1.16)	1.79 (1.44–2.21)	1.63
CD4 missing	1.06 (0.98–1.15)	2.69 (2.08–3.48)	2.53
HIV-negative	1 (reference)	1 (reference)	–
Known potential confounders			
Sex			
Female	1 (reference)	1 (reference)	–
Male	1.27 (1.23–1.31)	1.20 (1.06–1.37)	0.95
Age groups			
0–14	0.57 (0.51–0.63)	0.44 (0.25–0.77)	0.78
15–24	0.82 (0.78–0.87)	1.03 (0.84–1.26)	1.25
25–34	0.94 (0.90–0.98)	1.10 (0.94–1.28)	1.17
35–44	1 (reference)	1 (reference)	–
45–54	1.14 (1.08–1.20)	0.88 (0.72–1.08)	0.77
55+	0.94 (0.88–1.01)	0.68 (0.51–0.92)	0.72
Cumulative admission time^a^			
No admission	1 (reference)	1 (reference)	–
<1 week	2.03 (1.93–2.14)	1.69 (1.38–2.07)	0.83
≥1 week	3.44 (3.24–3.64)	3.93 (3.25–4.74)	1.14
Constant	0.007 (0.0068–0.0076)	0.0003 (0.0002–0.0003)	0.04
gamma	−0.06 (−0.064 to −0.057)	−0.049 (−0.061 to −0.037)	0.82

Abbreviations: Human immunodeficiency virus (HIV); cluster of differentiation 4 (CD4); hazard ratio (HR); confidence interval (CI); rifampicin-resistant TB (RR-TB); rifampicin-susceptible TB (RS-TB).

a Within either subsequent MDR/RR-TB diagnosis, censoring or 18 months (if no subsequent TB diagnosis) of TB outcome date.

## Discussion

These data show a substantially increased risk of subsequent confirmed MDR/RR-TB following first-line TB treatment initiation among PLHIV compared to those who are HIV-negative, exceeding the modest increased risk in DS-TB recurrence for PLHIV. While this increased risk could be due to either increased resistance-acquisition during treatment or increased MDR/RR-TB transmission, the independent effects of advanced HIV and hospitalization suggest that both mechanisms may be contributory. The increased risk of MDR/RR-TB among PLHIV is further supported by an increased MDR/RR-TB risk relative to the risk of any other diagnosed TB following successful TB treatment.

Increased TB drug resistance-acquisition during treatment among PLHIV would imply that current first-line TB treatment regimens may be inadequate for a significant proportion of people with TB, particularly those with advanced HIV. This finding is especially relevant with the introduction of shorter TB treatment regimens which may be less robust with real-world levels of treatment adherence.^16^^,^^17^ Unfortunately, clinical trials of both established and newer shorter TB regimens have not been, and are unlikely to be, powered to detect resistance-acquisition, with less than 1% of individuals commonly developing rifampicin-resistance during well-supported TB treatment.^17^ Therefore, large scale epidemiological studies using routine programmatic data, such as this one, are important for demonstrating relatively small effects at an individual level, but with potentially large and significant impacts at a population level.

While systematic reviews have shown associations between HIV and MDR/RR-TB overall, associations among new, treatment-naive individuals (suggesting transmission) and among those with prior first-line TB treatment (suggesting resistance-acquisition) have been inconsistent.^3^^,^^6^^,^^7^ These inconsistences may be due to a range of factors, including the relatively small and underpowered population sizes included in most studies, varying risks of differential exposure to MDR/RR-TB among PLHIV across different settings, and distinct study populations (e.g. hospitalized individuals, prisoners). In addition, MDR/RR-TB was often a secondary outcome, with few reported events in most studies.

TB drug resistance arises initially through the selection of spontaneously emerging resistance-conferring mutations under treatment pressure–hence the need for multidrug therapy for TB.^18^^,^^19^ However, even with multidrug therapy, bacterial populations may be exposed to single drugs (functional monotherapy) through a range of mechanisms, including: poor adherence to selective drugs, drug malabsorption, inadequate dosing or sub-standard drug quality.^5^^,^^18^ The increased MDR/RR-TB risk among people with advanced HIV disease shown here is consistent with data from small case series, predominantly drawn from clinical trials, which have identified low CD4 cell counts (less than 100 cells/μl) at TB treatment start as an important risk factor for the subsequent emergence of MDR/RR-TB.^9^ Given that gastrointestinal impacts in advanced HIV are common, malabsorption of TB drugs, leading to functional monotherapy, is a potential mechanism for increased resistance-acquisition.^10^ However, a systematic review found no consistent effect of HIV infection on TB drug pharmacokinetics, but did find that low plasma concentrations of key first-line drugs, particularly rifampicin, were common in both PLHIV and HIV-negative individuals.^20^ Both the area under the curve (AUC) and maximum plasma concentration of TB drugs are associated with resistance emergence.^21^ This is consistent with observations of increased resistance-acquisition during thrice weekly treatment compared to daily TB treatment regimens.^9^ As a result, altered rifampicin pharmacokinetics and lower drug exposure due to HIV, particularly in advanced HIV, may contribute to increased MDR/RR-TB risk among PLHIV observed here.

There are other possible explanations for an increased acquired MDR/RR-TB risk in PLHIV. HIV is associated with a greater risk of extrapulmonary and disseminated TB, and therefore, often higher total body TB bacterial burden, even when respiratory specimens may be paucibacillary.^22^ Given that the likelihood of spontaneously emerging resistant bacteria increases with bacterial burden, including the risk of spontaneous emergence of more than one independent resistance conferring mutation, the risk of within-host emergence of multi-drug resistant bacteria may be increased, particularly in conjunction with functional monotherapy. There are also known drug-drug interactions between ART and rifampicin, with both efavirenz- and dolutegravir- (rolled out in South Africa in 2019) based regimens resulting in decreased rifampicin exposure.^23^ However, evidence on their impact on MDR/RR-TB emergence is limited. Additionally, people with low body weight, common in advanced HIV, often have lower TB drug exposures with the use of weight-based dosing, due to higher proportional fat-free mass.^24^

Rifampicin resistance is also more likely to emerge *de novo* during standard first-line treatment in the presence of isoniazid (INH) mono-resistance.^3^^,^^11^^,^^12^ With the use of rapid molecular tests for TB diagnosis and rifampicin DST (e.g. Xpert MTB/RIF), INH mono-resistance is often not detected routinely in many high burden settings and yet is reported to be as high as 12% of all diagnosed TB in South Africa.^25^ Available data suggest that PLHIV have a higher prevalence of INH mono-resistant TB compared to HIV-negative individuals in some settings, including the United States,^26^ but data are limited in high TB burden settings, including South Africa. As INH DST is rarely conducted for those with rifampicin-sensitive TB, the contribution of INH mono-resistance to the observed effects in this study could not be assessed. Finally, while poor treatment adherence has often been cited as a cause of TB drug resistance, there is very little evidence supporting this assertion. Drug resistance emerges despite excellent adherence in clinical trials,^9^ and *in vitro* studies have demonstrated that high levels of simulated poor adherence, which despite causing treatment failure, do not generate resistant bacterial populations.^5^ Differential treatment adherence by HIV status would therefore seem unlikely to explain our results.

We observed extremely high levels of hospitalization in the 6 months after TB treatment initiation; 32% among PLHIV and 18% among HIV-negative individuals. Hospitalization remained high even for those with a successful TB treatment outcome; 12% of PLHIV and 9% of HIV-negative individuals were admitted in the follow-up period after treatment success. A review of South African studies on multimorbidity highlighted high comorbidity levels in TB patients.^27^ Thus, high hospitalization rates in this study could reflect the presence of significant comorbidities requiring ongoing care or post-TB sequelae, or both. Regardless, these data reaffirm calls to provide more intensive support and care for individuals completing TB treatment.^16^

Overall, hospitalization was a significant contributor to subsequent MDR/RR-TB diagnosis, with those admitted for more than a week cumulatively having a threefold increased MDR/RR-TB risk compared to those not admitted, after accounting for advanced HIV. We assessed the potential impact of hospitalization as a marker of increased nosocomial transmission risk, given that both HIV and infectious MDR/RR-TB are likely to be co-located in health facilities. However, as hospitalization is likely to be confounded by both advanced HIV and TB treatment outcome, we conducted a subset analysis restricted to those with successful treatment only, stratified by HIV-status. Among these, those hospitalized for more than a week had about three times the risk of subsequent MDR/RR-TB diagnosis among HIV-negative individuals and over four times the risk among PLHIV, compared to those not hospitalized, suggesting that nosocomial transmission may be important.

Given that resistance-conferring mutations may entail a bacterial fitness cost, some resistant TB strains may be more successfully transmitted to PLHIV with weakened immunity.^28^ In the absence of sequence data to discern specific ribonucleic acid (RNA) polymerase (*rpoB*) mutations, we were unable to assess this possibility. However, more recent data suggest that the impact of lower bacterial fitness may be minimal in high MDR/RR-TB burden settings.^29^

While we assessed admission as a mechanism for increased exposure to MDR/RR-TB and subsequent transmission, hospital admission is also likely to be associated with comorbidities other than HIV, as shown by the increased risk among HIV-negative individuals hospitalized for longer. Comorbidities, such as diabetes, common in our population,^30^ may also effect the risk of acquired TB drug resistance, similar to that of HIV. Although data on diagnosed diabetes are available through the PHDC, given the extent of undiagnosed diabetes in the province, we did not include this variable in the analysis. Nonetheless, the effect of hospitalization, seen in these data, suggests that reinfection, or potentially super-infection with already MDR/RR-TB strains, may contribute to an increased risk of MDR/RR-TB in our population. This underscores the need to strengthen infection and prevention control measures.

There was also a significant effect of HIV (at all CD4 count levels) and hospitalization on the incidence of all other (non MDR/RR-TB) subsequent TB in individuals with successful treatment. This higher risk of recurrent TB in successfully treated PLHIV has been shown previously in high TB and HIV prevalence settings, reflecting continued increased TB risk among PLHIV, potentially due to reinfection or relapse with the same TB strain, or nosocomial transmission.^1^^,^^5^^,^^8^^,^^10^^,^^20^ Furthermore, South Africa's generalized HIV and TB epidemics characterized by widespread transmission in the general population, together with socio-economic and other unmeasured risk factors, may also contribute to the increased MDR/RR-TB risk.

As this was a retrospective study, limitations include reliance on an algorithm to link various data sources to individuals, and limited data to assess confounding (e.g. by comorbidities such as diabetes and heart disease). Although CD4 cell count was used as a marker of HIV disease severity, factors related to HIV care, such as ART interruptions, that might have resulted in advanced HIV disease throughout follow-up, were not able to be incorporated into the analysis. In addition, PLHIV are potentially more likely to be screened for TB as part of their ART care, with a TB nucleic acid amplification test (NAAT) that detects RR-TB status. Furthermore, TB hetero-resistance where both drug-susceptible and drug-resistant TB strains can coexist in individuals may be a contributor to MDR/RR-TB that is diagnosed after an initial rifampicin-susceptible TB diagnosis. Finally, we used hospitalization as a proxy for nosocomial exposure; there may also be nosocomial transmission of MDR/RR-TB in outpatient health facilities.

Nonetheless, previous studies using these data exchange have indicated high accuracy in linkage, particularly for both TB- and HIV-related data.^15^ Differential follow-up between PLHIV and HIV-negative individuals is another potential limitation, with PLHIV more likely to have regular contact with health services. However, a sensitivity analysis censoring at 2-years of follow-up instead of last contact showed no difference in results (data not shown).

Despite these limitations, the study has notable strengths. The cohort is extremely large and has been drawn from the public health sector in the WCP, representing approximately 95% of HIV and TB patients in the province.^15^ While the WCP has exceptionally high TB incidence, even by African standards, the findings are likely generalizable to other high TB and HIV burden settings.^1^

Despite the importance of recent and ongoing work to develop and implement new shorter TB regimens,^17^ the emergence of TB drug resistance during therapy cannot be overlooked. These data emphasize the need for clinical trials of newer regimens to enrol sufficient numbers of PLHIV, particularly those with advanced HIV. Concurrent pharmacokinetic studies to assess and potentially model MDR/RR-TB resistance-acquisition during treatment risk are also required. These data also add weight to calls for a more individualized approach to TB treatment, accounting for variations in clinical characteristics such as TB disease severity, HIV status, comorbidities, and individual factors such as body weight. While logistically challenging therapeutic drug monitoring would also be beneficial. Furthermore, a re-emphasis on respiratory infection control measures for TB, and other airborne infections, in health facilities is required.

In conclusion, these data show that PLHIV and individuals admitted to hospitals are at a substantially higher risk of MDR/RR-TB following first-line TB treatment initiation. Given that the individual risk of resistance-acquisition, although important, is relatively low, even small increases of resistance emergence will continue to fuel the MDR/RR-TB epidemic in high burden settings, such as South Africa, where over 200,000 individuals are treated for TB annually.

## Contributors

All authors contributed to conceptual design of this study and to drafting and revising of the final manuscript. Additionally, all authors have seen and approved the final manuscript. NZ and HC have verified the underlying data. All authors had full access to all the data in the study and had final responsibility for the decision to submit for publication.

## Data sharing statement

The de-identified individual level data in this study are under a data use agreement that does not permit public sharing. Processed data underlying specific figures and tables, including data not presented, are available upon reasonable request to the corresponding author.

## Declaration of interests

All authors declare no competing interests.
