# Supplementary material for: Impact of HIV and hospitalization on the incidence of subsequent rifampicin-resistant tuberculosis after initiation of first-line tuberculosis treatment: a retrospective cohort study in South Africa
Source: eClinicalMedicine. 2025 Oct 30;90:103603. doi: 10.1016/j.eclinm.2025.103603 (PMC12613042; doi:10.1016/j.eclinm.2025.103603)
Supplement: Supplementary Materials [file mmc1.docx]

**Supplementary Materials**

**Impact of HIV and hospitalization on the incidence of subsequent rifampicin-resistant tuberculosis after initiation of first-line tuberculosis treatment: a retrospective cohort study in South Africa**

**Table of Contents**

[Causal pathway summarizing the effect of HIV and hospitalization on subsequent MDR/RR-TB diagnosis 2](#_Toc209191384)

[Testing of the proportional hazards assumption of the Cox model 4](#_Toc209191385)

[Identifying the Parametric survival model 5](#_Toc209191386)

[References 9](#_Toc209191387)

**Figures**

**Figure S1:** Causal diagram summarizing the effect of HIV and hospitalization on subsequent MDR/RR-TB diagnosis……………………………………………………….2

**Figure S2:** Test of the proportional hazard assumption for sex, agegroup, CD4 cell count and cumulative admission time…………………………………………………………….5

**Figure S3**: Goodness of fit of selected Gompertz survivor model using cumulative hazard of Cox-Snell residuals……………………………………………………………………...7

**Tables**

**Table S1**: People living with human immunodeficiency virus (PLHIV) cohort (N=69,636) by CD4 cell count…………………………………………………………………………3

**Table S2:** Testing of the proportional hazard assumption…………….…………………...4

**Table S3:** Goodness of fit metrics: Akaike Information Criterion (AIC) and Bayesian Information Criterion (BIC)…………………………………………………………….…6

**Table S4:** Comparison of the parametric survival models…………………………………6

**Table S5:** Variance inflation factor (VIF)………………………………………………….8

# Causal pathway summarizing the effect of HIV and hospitalization on subsequent MDR/RR-TB diagnosis


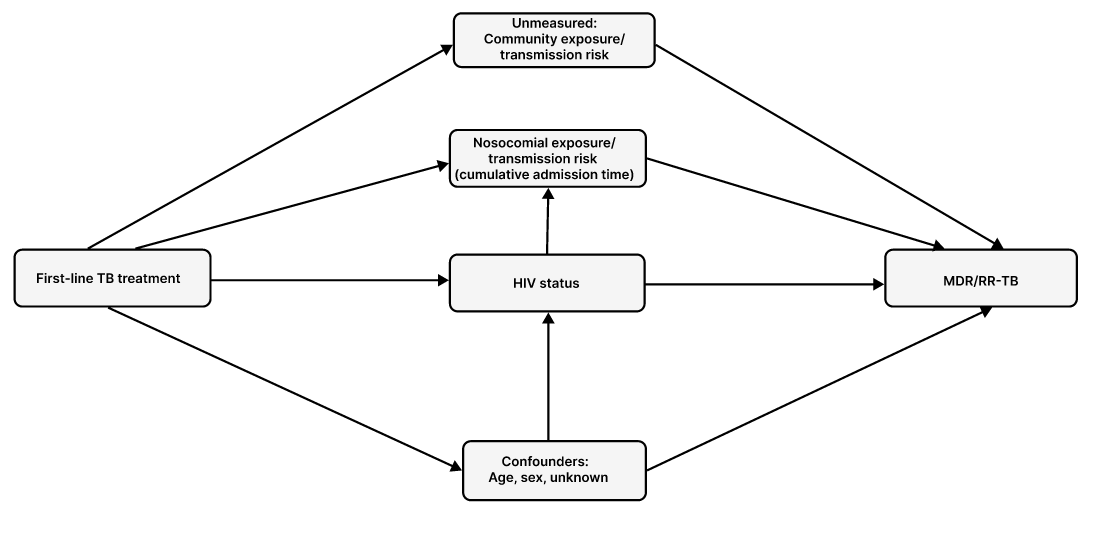


**Figure S1: Causal diagram summarizing the effect of human immunodeficiency virus (HIV) and hospitalization on subsequent multi-drug or rifampicin-resistant tuberculosis (MDR/RR-TB) diagnosis.**

Causal inference helps us understand how exposure affects an outcome.^1^ In our study in where all individuals initiated first-line tuberculosis (TB) treatment, the primary exposures were human immunodeficiency virus (HIV) (and HIV severity, assessed by cluster of differentiation 4 (CD4) cell count) and nosocomial transmission risk (cumulative admission time), with subsequent multi-drug or rifampicin-resistant tuberculosis (MDR/RR-TB) as the outcome. Confounders are variables that influence both the exposure and outcome, potentially biasing effect estimates and leading to incorrect conclusions.^2^ To control for this, we used regression adjustment guided by a causal diagram and prior knowledge. Confounders included age and sex, both of which are known to be associated with both HIV and MDR/RR-TB, along with other unknown (unmeasured) risk factors. Given that TB is a generalized epidemic in the country with widespread community exposure, we also included community transmission as a potential risk factor in the casual pathway. A causal diagram illustrating these relationships is presented in Figure S1.

**Table S1: People living with human immunodeficiency virus (PLHIV) cohort (N=69,636) by CD4 cell count**

|  | **CD4 cell count**  **<200** cells/µl | **CD4 cell count**  **>200** cells/µl | **No CD4** |
| --- | --- | --- | --- |
| **Total** | 38191 | 22098 | 9347 |
| **Female** | 17308 (45·3) | 11776 (53·3) | 5009 (53·6) |
| **Male** | 20883 (54·7) | 10322 (46·7) | 4338 (46·4) |
| **Agegroup** |  |  |  |
| **0-14** | 199 (0·5) | 515 (2·3) | 132 (1·4) |
| **15-24** | 2852 (7·5) | 2903 (13·1) | 954 (10·2) |
| **25-34** | 15263 (40·0) | 8363 (37·8) | 3139 (33·6) |
| **35-44** | 13310 (34·9) | 6369 (28·8) | 3043 (32·6) |
| **45-54** | 5107 (13·4) | 2950 (13·3) | 1462 (15·6) |
| **55+** | 1460 (3·8) | 998 (4·5) | 617 (6·6) |
| **TB treatment district** |  |  |  |
| **Cape Town metro** | 25924 (67·9) | 14391 (65·1) | 5899 (63·1) |
| **Other districts*** | 12267 (32·1) | 7707 (34·9) | 3448 (36·9) |

******Includes Cape Winelands, Central Karoo, Garden Route, Overberg and West Coast districts.* Abbreviations: Cluster of differentiation (CD4).

# Testing of the proportional hazards assumption of the Cox model

**Table S2: Testing of the proportional hazard assumption**

|  | **Rho** | **Chi2** | **Prob>chi2** |
| --- | --- | --- | --- |
| **Sex** | 0·021 | 1·14 | 0·285 |
| **Agegroup** | 0·061 | 7·71 | 0·006 |
| **Cumulative admission time** | 0·197 | 96·01 | 0·000 |
| **CD4 cell count** | -0·146 | 54·17 | 0·000 |
| **Global test** |  | 131·58 | 0·000 |

Initially, Cox regression models were used to assess the impact of HIV (and HIV severity, assessed by CD4 cell count) on the incidence of subsequent MDR/RR-TB (DST) from the timepoint of first-line TB treatment initiation. However, the proportional hazard assumption was violated based on the Schoenfeld and scaled Schoenfeld residuals the proportional hazard assumption was violated (Table S2 and Figure S2), thus used parametric survival models as an alternative.

**Figure S2: Test of the proportional hazard assumption for sex, agegroup, cluster of differentiation 4 (CD4) cell count and cumulative admission time**

# Identifying the Parametric survival model

Parametric survival models assume that the survival times follow a specified probability distribution, with parameters of the distribution depending on covariates.^3^ Parametric survival regression models including the Exponential, Weibull, Log-normal, Log-logistic and the Gompertz distributions were fitted. We used the goodness of fit metrics Akaike Information Criterion (AIC) and the Bayesian Information Criterion (BIC) to identify the best model fit, with lower values of AIC and BIC indicating the better fit. Based on the lowest values of AIC and BIC values, the Gompertz distribution was identified as the best fit (Table S3). A comparison of the fitted parametric models is presented in Table S4.

**Table S3: Goodness of fit metrics: Akaike Information Criterion (AIC) and Bayesian Information Criterion (BIC)**

| **Model** | **AIC** | **BIC** |
| --- | --- | --- |
| Cox | 60428·20 | 60539·96 |
| Exponential | 31583·77 | 31705·68 |
| Weibull | 31534·79 | 31666·86 |
| Log-normal | 31429·14 | 31561·22 |
| Log-logistic | 31528·18 | 31660·25 |
| Gompertz | 31415·83 | 31547·91 |

**Table S4: Comparison of the parametric survival models**

|  | **Cox** | **Exponential** | **Weibull** | **Log-normal** | **Log-logistic** | **Gompertz** |
| --- | --- | --- | --- | --- | --- | --- |
|  | **aHR (95% CI)** | **aHR (95% CI)** | **aHR (95% CI)** | **Coef (95% CI)** | **Coef (95% CI)** | **aHR (95% CI)** |
| **CD4 cell count ≤ ±6 months of treatment start** |  |  |  |  |  |  |
| **0-199** | 2·86 (2·60 - 3·15) | 2·88 (2·61 - 3·17) | 2·87 (2·60 - 3·16) | -1·22 (-1·34 - -1·09) | -1·21 (-1·33 - -1·09) | 2·86 (2·60 - 3·15) |
| **200+** | 1·38 (1·20 - 1·58) | 1·38 (1·20 - 1·58) | 1·38 (1·20 - 1·58) | -0·33 (-0·49 - -0·17) | -0·37 (-0·52 - -0·21) | 1·38 (1·20 - 1·58) |
| **CD4 missing** | 2·02 (1·71 - 2·39) | 2·02 (1·71 - 2·40) | 2·02 (1·71 - 2·40) | -0·78 (-0·99 - -0·58) | -0·81 (-1·00 - -0·61) | 2·02 (1·71 - 2·39) |
| **HIV-negative** | 1 (reference) | 1 (reference) | 1 (reference) | 1 (reference) | 1 (reference) | 1 (reference) |
| **Sex** |  |  |  |  |  |  |
| **Female** | 1 (reference) | 1 (reference) | 1 (reference) | 1 (reference) | 1 (reference) | 1 (reference) |
| **Male** | 1·10 (1·01 - 1·19) | 1·10 (1·01 - 1·19) | 1·10 (1·01 - 1·19) | -0·11 (-0·21 - -0·01) | -0·11 (-0·20 - -0·02) | 1·10 (1·01 - 1·19) |
| **Age groups** |  |  |  |  |  |  |
| **0-14** | 0·61 (0·47 - 0·78) | 0·60 (0·47 - 0·78) | 0·61 (0·47 - 0·78) | 0·62 (0·33 - 0·91) | 0·58 (0·28 - 0·87) | 0·61 (0·47 - 0·79) |
| **15-24** | 0·97 (0·85 - 1·10) | 0·97 (0·85 - 1·10) | 0·97 (0·85 - 1·10) | 0·11 (-0·05 - 0·26) | 0·04 (-0·11 - 0·19) | 0·97 (0·85 - 1·10) |
| **25-34** | 1·04 (0·94 - 1·15) | 1·04 (0·94 - 1·15) | 1·04 (0·94 - 1·15) | -0·02 (-0·15 - 0·10) | -0·04 (-0·16 - 0·07) | 1·04 (0·94 - 1·15) |
| **35-44** | 1 (reference) | 1 (reference) | 1 (reference) | 1 (reference) | 1 (reference) | 1 (reference) |
| **45-54** | 0·86 (0·75 - 0·98) | 0·86 (0·75 - 0·98) | 0·86 (0·75 - 0·98) | 0·17 (0·01 - 0·33) | 0·18 (0·03 - 0·33) | 0·85 (0·75 - 0·98) |
| **55+** | 0·63 (0·52 - 0·76) | 0·63 (0·53 - 0·76) | 0·63 (0·52 - 0·76) | 0·50 (0·29 - 0·71) | 0·53 (0·31 - 0·74) | 0·63 (0·52 - 0·76) |
| **Cumulative admission time*** |  |  |  |  |  |  |
| **No admission** | 1 (reference) | 1 (reference) | 1 (reference) | 1 (reference) | 1 (reference) | 1 (reference) |
| **<1 Week** | 1·21 (1·08 - 1·36) | 1·21 (1·08 - 1·36) | 1·21 (1·08 - 1·36) | -0·22 (-0·36 - -0·08) | -0·22 (-0·35 - -0·08) | 1·21 (1·07 - 1·36) |
| **≥ 1 Week** | 2·76 (2·50 - 3·05) | 2·79 (2·52 - 3·08) | 2·76 (2·50 - 3·05) | -1·41 (-1·55 - -1·28) | -1·18 (-1·30 - -1·06) | 2·75 (2·49 - 3·04) |
| **Constant** | - | 0·00 (0·00 - 0·00)  0·000333 | 0·00 (0·00 - 0·00)  0·000497 | 10·57 (10·27 - 10·86) | 8·67 (8·42 - 8·92) | 0·00 (0·00 - 0·00)  0·000504 |
| **p** |  |  | 0·87 (0·84 - 0·91) |  |  |  |
| **sigma** |  |  |  | 3·08 (2·97 - 3·19) |  |  |
| **gamma** |  |  |  |  | 1·14 (1·10 - 1·18) |  |
| **/gamma** |  |  |  |  |  | -0·04 (-0·04 - -0·03) |

Abbreviations: Adjusted hazard ratio(aHR); Confidence interval (CI); Cluster of differentiation 4 (CD4); Human immunodeficiency virus (HIV).

**Figure S3: Goodness of fit of selected Gompertz survivor model using cumulative hazard of Cox-Snell residuals**

We assessed the goodness of fit of the selected Gompertz model using the Cox-Snell residuals, by plotting the Nelson-Aalen cumulative hazard against the residuals. As shown in Figure S3, there is general alignment between the model’s fitted hazards and the observed data, suggesting Gompertz survival model is a good fit.

**Table S5: Variance inflation factor (VIF)**

|  | **VIF** | **1/VIF** |
| --- | --- | --- |
| **CD4 cell count ≤ ±6 months of treatment start** |  |  |
| **0-199** | 1·35 | 0·738 |
| **200+** | 1·13 | 0·886 |
| **CD4 missing** | 1·05 | 0·955 |
| **HIV-negative** | 1 (reference) | 1 (reference) |
| **Sex** |  |  |
| **Female** | 1 (reference) | 1 (reference) |
| **Male** | 1·82 | 0·550 |
| **Age groups** |  |  |
| **0-14** | 1·08 | 0·926 |
| **15-24** | 1·18 | 0·851 |
| **25-34** | 1·48 | 0·674 |
| **35-44** | 1 (reference) | 1 (reference) |
| **45-54** | 1·24 | 0·807 |
| **55+** | 1·16 | 0·863 |
| **Cumulative admission time*** |  |  |
| **No admission** | 1 (reference) | 1 (reference) |
| **<1 Week** | 1·20 | 0·833 |
| **≥ 1 Week** | 1·19 | 0·838 |
| **Mean VIF** | **1·26** |  |

Abbreviations: Cluster of differentiation 4 (CD4); Human immunodeficiency virus (HIV).

The variance inflation factor (VIF) for all the risk factors ranges between 1 and 2, indicating no significant multicollinearity that requires correction.

# References

1 VanderWeele TJ, Chiba Y· Sensitivity analysis for direct and indirect effects in the presence of exposure-induced mediator-outcome confounders· *Epidemiology, Biostatistics, and Public Health* 2014; **11**· DOI:10·2427/9027·

2 Westreich D, Greenland S· The Table 2 Fallacy: Presenting and Interpreting Confounder and Modifier Coefficients· *Am J Epidemiol* 2013; **177**: 292–8·

3 Cleves MA, Gould W, Marchenko YV· An Introduction to Survival Analysis Using Stata· Stata Press, 2016 https://books·google·co·za/books?id=0FH1jwEACAAJ·
